# Comparative Analysis of Gut Microbiome Community Structures in Different Populations of Asian Elephants in China and Their Correlation with Diet

**DOI:** 10.3390/genes16050483

**Published:** 2025-04-25

**Authors:** Qiang Guo, Wenping Zhang, Chenyang Xu, Xu Li, Bin Wang, Chaoyong Xiong, Wenguang Duan, Tingting Luo, Weibin Wang, Jielong Zhou

**Affiliations:** 1 Key Laboratory of Conserving Wildlife with Small Populations in Yunnan/College of Biological Science and Food Engineering, Southwest Forestry University, Kunming 650224, China; guoq9382@163.com (Q.G.); 18879801146@163.com (C.X.); lixu.swfu@hotmail.com (X.L.); 2 Key Laboratory of Forest Resources Conservation and Utilization in the Southwest Mountains of China, Ministry of Education, Southwest Forestry University, Kunming 650224, China; 3 Key Laboratory of Monitoring Biological Diversity in Minshan Mountain of National Park of Giant Pandas at Mianyang Teachers’ College of Sichuan Province, College of Life Science and Biotechnology, Mianyang Normal University, Mianyang 621000, China; zhang_zoology@163.com; 4 Management and Protection Bureau of Yunnan Xishuangbanna National Nature Reserve, Xishuangbanna 666100, China; tree3@163.com (B.W.); 18988133909@163.com (C.X.); 13808708664@163.com (T.L.); 5 Management and Protection Bureau of Yunnan Nangunhe National Nature Reserve, Lincang 650223, China; 18787494548@139.com

**Keywords:** Asian elephant, gut microbiome, metagenomic sequencing, microbial diversity, diet–gut microbiome interaction

## Abstract

Background: The interaction and co-evolution between the gut microbiome and the host play important roles in the host’s physiology, nutrition, and health. Diet is considered an important driver of differences in the gut microbiota; however, research on the relationship between the gut microbiota and diet in Asian elephants remains limited. Methods: In this study, we explored the gut microbiota structure and its relationship with diet in different populations of Asian elephants through metagenomic sequencing, combined with previously published dietary data. Results: This study found that the dominant gut microbiota of Asian elephants includes the phyla Bacillota (29.85% in BP, 22.79% in RC, 21.89% in SM, 31.67% in ML, and 33.00% in NGH), Bacteroidota (25.25% in BP, 31.44% in RC, 16.44% in SM, 25.73% in ML, and 23.74% in NGH), and Spirochaetota (3.49% in BP, 6.18% in RC, 1.71% in SM, 2.69% in ML, and 3.52% in NGH), with significant differences in the gut microbiota among different populations. Correlation analysis between the gut microbiota and diet revealed that dietary diversity did not directly affect the alpha diversity of the gut microbiota. However, specific food types might play a key role in shaping the gut microbiota structure by regulating the abundance of certain microbiota. Conclusions: This study reveals significant differences in the gut microbiota structure among different populations of Asian elephants and explores the impact of diet on the structure. The results provide foundational data for a deeper understanding of the gut microbiota structure of Asian elephants and offer important references for the scientific conservation and precise management strategies of this species.

## 1. Introduction

The Asian elephant (*Elephas maximus*) is the largest terrestrial vertebrate in Asia, an ecosystem engineer, and a flagship species, playing an essential role in maintaining ecosystem integrity [1,2]. Additionally, elephants hold significant symbolic value in numerous traditional cultures [3]. Due to threats such as habitat fragmentation and poaching, the Asian elephant has been listed as an Endangered (EN) species on the International Union for Conservation of Nature (IUCN) Red List since 1986 [4]. Today, only approximately 300 wild Asian elephants in China are extant, distributed across Xishuangbanna, Pu’er, and Lincang in Yunnan Province, and they are classified into four genetic populations, including the Banna–Pu’er (BP), Shangyong–Mengla (SM), Menghai–Lancang (ML), and Nangunhe (NGH) populations [5].

The Asian elephant is a typical hindgut fermenter that depends on trillions of gut microbiota within its gastrointestinal tract to ferment food and obtain nutrients essential for sustaining its life activities [6,7,8]. The complex intestinal microbial communities play a critical role in host health maintenance, ecological adaptation, and disease prevention [9]. However, the gut microbiota is a complex and dynamic ecosystem influenced by a multitude of factors, including dietary habits, phylogenetic background, geographical location, age, and environmental conditions [10,11,12]. Among these factors, diet is one of the most critical, as the type and composition of food directly influence the structure and function of the gut microbiota [13].

An increasing number of studies have suggested that dietary changes can influence the gut microbiota structure of herbivorous wildlife [14]. However, studies on large herbivores, such as the Asian elephant, remain limited, hindering our understanding of the interaction between diet and the gut microbiota. This knowledge gap may impact the assessment of the health, management, and conservation strategies of Asian elephants. Therefore, investigating the response of the gut microbiota to different food sources is crucial for understanding the adaptability of their gut microbiota and providing scientific support for habitat management and captive breeding. In wildlife research, linking plant DNA metabarcoding data from fecal samples with gut microbiome data is an important approach to studying the interaction between diet and the gut microbiota and has been widely applied in species such as the African buffalo (*Syncerus caffer*) and wild gibbons (*Hoolock tianxing*) [14,15,16,17].

In this study, we collected fecal samples from different wild and rescued populations of Asian elephants in China and performed metagenomic sequencing to explore the gut microbiota structure of these populations. By integrating dietary metabarcoding data and conducting correlation analysis, we further investigated the impact of diet on the gut microbiota. This research provides important foundational data for understanding the gut microbiota structure of Asian elephants in China and offers scientific evidence for the development of health management and scientific feeding strategies for this species.

## 2. Materials and Methods

### 2.1. Dung Sample Collection

**Figure 1:**
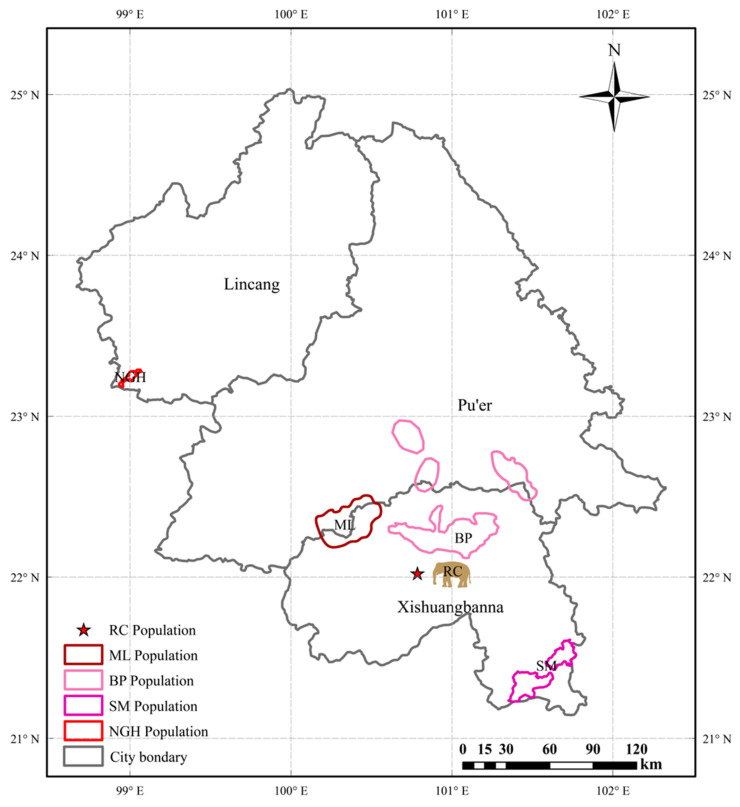
The locations of Asian elephant fecal sample collection in Yunnan Province, China. The five-pointed star marker represents the Xishuangbanna Asian Elephant Rescue and Breeding Center population (RC, *n* = 11). The areas marked with different colors indicate the distribution ranges of various wild populations. BP: Banna–Pu’er population (*n* = 60); SM: Shangyong–Mengla population (*n* = 8); ML: Menghai–Lancang population (*n* = 9); NGH: Nangunhe population (*n* = 19).

The fecal samples of Asian elephants in this study were collected from different wild elephant populations in Xishuangbanna Dai Autonomous Prefecture, Pu’er City, and Lincang City of Yunnan Province, China, as well as from captive elephants at the Xishuangbanna Asian Elephant Rescue and Breeding Center (Figure 1). From January to April 2023, fresh dung samples were collected from different populations, immediately frozen after collection, and transported to the laboratory for individual identification using microsatellite markers and mitochondrial DNA. In total, 107 individual samples were identified for use in this study, with detailed information provided in Supplementary Table S1. Based on geographical population classification, 60 samples were from the Banna–Pu’er (BP) population, 8 were from the Shangyong–Mengla (SM) population, 9 were from the Menghai–Lancang (ML) population, 19 were from the Nangunhe (NGH) population, and 11 were from the Xishuangbanna Asian Elephant Rescue and Breeding Center (RC) population (Figure 1 and Supplementary Table S1).

### 2.2. Microbial DNA Extraction and Metagenomic Sequencing

The total DNA was extracted from dung samples of Asian elephants using the QIAamp DNA Stool Mini Kit (Qiagen, Düsseldorf, Germany) following the manufacturer’s instructions. The quality and integrity of the extracted DNA were verified by electrophoresis on a 1% agarose gel, and concentration and purity were assessed using a NanoDrop2000 spectrophotometer (Thermo Fisher Scientific, Waltham, MA, USA). High-quality DNA samples were utilized for constructing metagenomic libraries with the NEB Next^®^ Ultra™ II DNA Library Prep Kit for Illumina^®^ (New England Biolabs, Ipswich, MA, USA). Qualified libraries were subjected to paired-end sequencing by Novogene (Beijing, China) on the Illumina NovaSeq X plus platform.

### 2.3. Metagenomic Sequence Analysis

Raw reads were first trimmed using Trimmomatic (Version 0.39) [18] to remove low-quality data. Subsequently, bowtie2 (Version 2.5.2) [19] was used to map the raw reads to the human genome (GCA_009914755.4) and the Asian elephant genome (GCA_033060105.1) to remove potential DNA contamination and produce clean reads. The clean reads were assembled using MEGAHIT (Version 1.2.9) [20]. The resulting scaffolds were broken at N junctions, and fragments shorter than 500 bp were filtered out to produce scaftigs [20]. Prodigal (Version 2.6.3) [21] was used for the prediction of open reading frames (ORFs) from scaftigs, and ORFs < 100 bp were filtered out. CD-HIT (Version 4.8.1) [22] was used to eliminate redundancy and obtain the non-redundant initial gene catalogue. Clean reads from each sample were aligned to the initial gene catalogue using Bowtie2, and read counts for each gene were quantified per sample [23]. Genes with read counts ≤ 2 were excluded to construct the Unigenes sequences. The abundance of each gene in each sample was calculated based on the number of aligned reads and the gene length.

DIAMOND (Version 2.1.9) [24] was used to align the Unigenes sequences with the Micro_NR database, providing taxonomic information on bacteria, fungi, archaea, and viruses. The alpha diversity indices, including the Shannon index, Simpson index, and Chao1 index, were calculated based on species abundance data, and the Kruskal–Wallis H test was used to assess the differences in the alpha diversity among different populations. The beta diversity among populations was analyzed using Principal Coordinate Analysis (PCoA) and Nonmetric Multidimensional Scaling (NMDS) based on the Bray–Curtis distance matrix. At the same time, based on the Bray–Curtis distance matrix, PERMANOVA (permutational multivariate analysis of variance) with 9999 permutations was performed to assess the significant differences in the microbial community composition between different groups.

### 2.4. Dietary Metabarcoding Sequencing and Analysis

We extracted the total DNA from fecal samples using the DNeasy Plant Pro Kit (Qiagen, Germany). Using the extracted DNA as a template, the pair primers rbcL2_F (5′-YGATGGACTTACNAGTCTTGATCGTTACA-3′) and rbcL2_R (5′-GNCCATAYTTRTTCAATTTATCTCTTTCAACTTGGATNCC-3′) were used to amplify the chloroplast rbcL gene region (280 bp). For the polymerase chain reaction (PCR), we used 15 µL Phusion High-Fidelity PCR Master Mix, 0.2 μM primers, and 10 ng of DNA template and adjusted the reaction volume to 25 µL with ddH_2_O. The PCR amplification program was set to an initial denaturation temperature of 98 °C for 1 min, followed by 30 cycles of denaturation at 98 °C for 10 s, annealing at 50 °C for 30 s, extension at 72 °C for 30 s, and a final extension at 72 °C for 5 min. The PCR products were analyzed by electrophoresis on a 2% agarose gel. Qualified PCR products were purified using magnetic beads and quantified with a Qubit 3.0 fluorometer (Life Technologies, Fremont, CA, USA), followed by equimolar pooling. After constructing the DNA library, the qualified libraries were paired-end sequenced on the Illumina NovaSeq 6000 platform by Novogene (Beijing, China). After sequencing, the sample data were demultiplexed based on the barcode and PCR primer sequences, and both the barcode and primer sequences were removed. FLASH (version 1.2.11) was used to merge the reads from each sample, generating raw reads [25]. To avoid interference in subsequent analyses, the reverse primer sequences were matched and trimmed using Cutadapt (version 4.4) [26]. Quality control of the raw reads was performed using fastp (version 0.23.2) [27], with low-quality reads filtered out to obtain high-quality clean data. The DADA2 plugin within QIIME2 (Version 2023.8) was used for denoising and chimera removal [28], generating high-resolution amplicon sequence variants (ASVs) and a feature table. To identify the taxonomic information of plant sequences in the samples, rbcL sequences were downloaded from GenBank and the Barcode of Life Data System (BOLD) and combined with a local plant list to build a local DNA reference database. Using QIIME2, the ASVs were compared against the local DNA reference database to annotate the species for each ASV (Supplementary Tables S2–S4).

### 2.5. Diet–Gut Microbiome Correlation Analysis

Procrustes analysis was performed to assess the concordance between the dietary composition and gut microbiota composition. Pearson correlation analysis was used to examine the relationship between the gut microbiota and dietary traits, while the Mantel test was conducted to evaluate the potential association between diet and gut microbial communities. All analyses were conducted in R, with scripts adapted from previously published studies [29,30,31].

### 2.6. Statistical Analysis

The experimental data are expressed as the mean ± SD. Kruskal–Wallis H tests were performed to compare the differences in alpha and beta diversity among different populations; * *p* < 0.05, ** *p* < 0.01, and *** *p* < 0.001.

## 3. Results

### 3.1. Fecal Metagenomic Sequencing and Assembly

In total, 1341 gigabases (Gb) of raw reads was obtained from the 107 fecal samples by shotgun metagenomic sequencing, with an average of 12.5 Gb (ranging from 9.86 to 21.02 Gb) for each sample (Supplementary Table S5). After filtering out low-quality and host reads, a total of 1325.32 Gbase of high-quality reads was obtained for assembly (Supplementary Table S5). The average total length, number of scaftigs, length, and N50 length were 836,573,636 bp, 729,662, 1159 bp, and 1257 bp, respectively (Supplementary Table S6). The gene prediction and abundance analysis revealed that the total number of non-redundant genes, total gene length, and average gene length were 28,395,179, 17,635.03 Mbp, and 621.06 bp, respectively.

### 3.2. The Gut Microbiota Profile of the Asian Elephant in China

**Figure 2:**
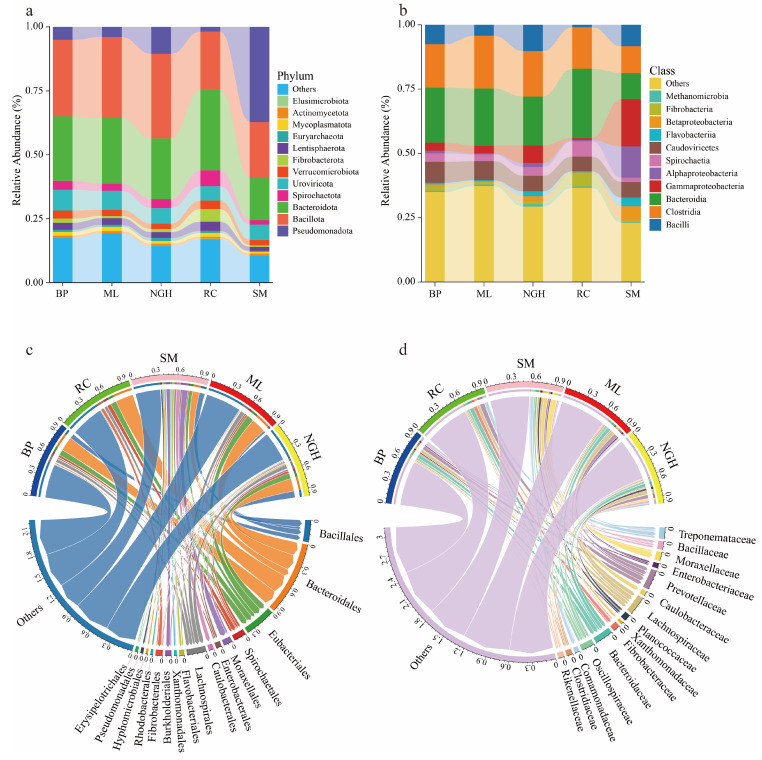
Gut microbiome composition of Asian elephant across different populations in China. (**a**) The stacked bar plot shows the relative abundance of the main gut microbiota at the phylum level within each group. Different colors represent different phyla, and lower-abundance taxa are grouped together as “Others”. (**b**) The stacked bar plot shows the relative abundance of the main gut microbiota at the class level within each group. Different colors represent different classes, and lower-abundance taxa are grouped together as “Others”. (**c**) The Chord diagram displays the relative abundance of the main gut microbiota at the order level within each group. The width of the connecting bars indicates the relative abundance of the microbiota at the order level across different groups, with lower-abundance taxa grouped as “Others”. (**d**) The Chord diagram displays the relative abundance of the main gut microbiota at the family level within each group. The width of the connecting bars indicates the relative abundance of the microbiota at the family level across different groups, with lower-abundance taxa grouped as “Others”.

At the phylum level, Bacillota, Bacteroidota, and Pseudomonadota were the dominant phyla, which were present in all samples as core microbiome components (Figure 2a and Supplementary Table S7). In SM, Pseudomonadota dominated with a relative abundance of 37.22%, whereas its relative abundance was lower in BP, ML, NGH, and RC, at 5.08%, 1.82%, 3.94%, and 10.58%, respectively. Bacillota and Bacteroidota were the dominant phyla across all groups, accounting for a high proportion in each. Specifically, their relative abundances were 29.85% and 25.25% in BP, 31.67% and 25.73% in ML, 33.00% and 23.74% in NGH, 22.79% and 31.44% in RC, and 21.89% and 16.44% in SM, respectively (Figure 2a and Supplementary Table S7).

At the class level, Clostridia and Bacteroidia were the dominant microbes, with relative abundances of 16.88% and 21.37% in BP, 16.19% and 26.95% in RC, 10.38% and 10.14% in SM, 20.48% and 22.24% in ML, and 17.67% and 18.98% in NGH, respectively (Figure 2b and Supplementary Table S8). Interestingly, Gammaproteobacteria were dominant in SM, with a relative abundance of 18.46%, significantly higher than in other populations. Notably, Alphaproteobacteria were almost exclusively present in SM, with a relative abundance of 12.16%, whereas their abundance in the other populations was around 1%. Caudoviricetes, as a type of bacteriophage, was present in all populations (Figure 2b and Supplementary Table S8).

At the order level, Bacteroidales and Eubacteriales were the dominant microbes, with relative abundances of 20.43% and 8.45% in BP, 25.69% and 8.47% in RC, 9.74% and 5.05% in SM, 21.31% and 10.21% in ML, and 18.17% and 8.18% in NGH, respectively (Figure 2c and Supplementary Table S9). Bacillales was widely present in all wild populations but was barely detected in the captive population (relative abundance 0.55%). Xanthomonadales, Caulobacterales, Flavobacteriales, and Moraxellales exhibited relatively high abundance in SM, while their abundances were low in other populations (Figure 2c and Supplementary Table S9).

At the family level, Lachnospiraceae, Prevotellaceae, Treponemataceae, and Bacteroidaceae were the predominant families (Figure 2d and Supplementary Table S10). The relative abundance of Bacillaceae in wild populations was 3.05% in BP, 2.60% in SM, 1.17% in ML, and 3.56% in NGH, whereas it was only 0.22% in the captive population (RC group). Caulobacteraceae (5.74%), Xanthomonadaceae (2.60%), and Pseudomonadaceae (2.54%) were primarily enriched in SM, while their relative abundances remained extremely low (<0.90%) in other populations. The relative abundance of Moraxellaceae was 8.69% in SM and 2.51% in NGH, while it remained below 0.4% in the other populations (Figure 2d and Supplementary Table S10).

### 3.3. Alpha and Beta Diversity of Gut Microbiome Across Different Populations

**Figure 3:**
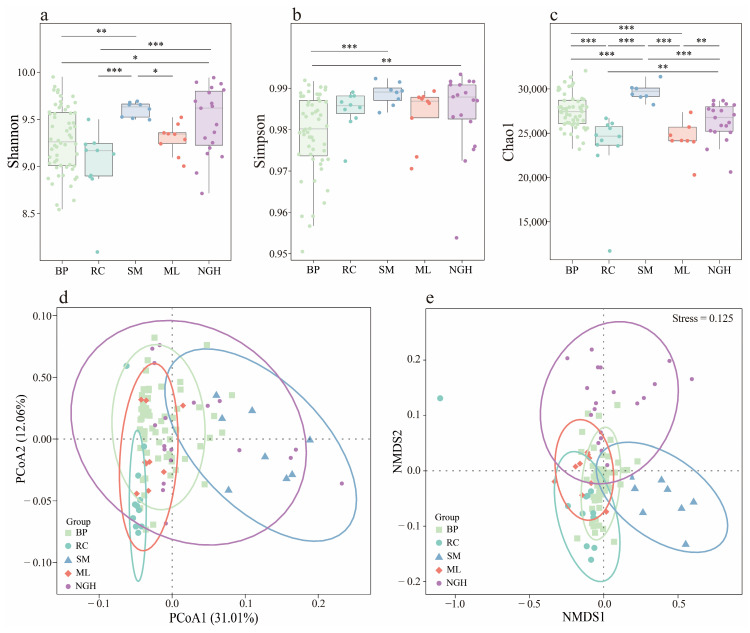
Gut microbiome diversity of Asian elephant across different populations in China. (**a**–**c**) Box plots display the comparison of alpha diversity differences in the gut microbiota composition across different populations, with (**a**) showing the Shannon index, (**b**) showing the Simpson index, and (**c**) showing the Chao1 index. Significant differences detected by the Kruskal–Wallis H test; “*” indicates significant difference between two groups; * *p* < 0.05, ** *p* < 0.01, and *** *p* < 0.001. (**d**) Principal Coordinate Analysis (PCoA) of the beta diversity in the gut microbiota composition across different populations based on the Bray–Curtis distance. (**e**) Nonmetric Multidimensional Scaling (NMDS) analysis of the beta diversity in the gut microbiota composition across different populations based on the Bray–Curtis distance.

The alpha diversity indicates intergroup differences in the gut microbiome diversity of Asian elephants across different areas (Figure 3a–c). The Shannon index shows that SM and NGH have the highest diversity, with no significant difference between them. Similarly, there is no significant difference between RC, BP, and ML (*p* > 0.05), while significant differences are observed between the remaining groups (Figure 3a). The Simpson index shows that only BP exhibits significant differences with SM and NGH, while no significant differences are observed between the other groups (Figure 3b). The Chao1 index analysis indicates that SM exhibits the highest richness, while RC exhibits the lowest. No significant differences were observed between BP and NGH and RC and ML, while significant differences were observed between all other pairs (Figure 3c).

Principal Coordinate Analysis (PCoA) and Nonmetric Multidimensional Scaling (NMDS) based on the Bray–Curtis distance for beta diversity analysis revealed significant differences in the gut microbiota composition of Asian elephants across different regions (Figure 3d,e). Notably, the PCoA and NMDS plot illustrated the similarity in microbial communities between BP and ML while highlighting the differences between RC and SM. To further validate these results, we performed a PERMANOVA on the Bray–Curtis distance matrix. The results of the PERMANOVA showed significant differences in the gut microbiome composition between most groups (Table 1). Notably, the differences between SM and ML (R^2^ = 0.464), RC and SM (R^2^ = 0.453), and BP and SM (R^2^ = 0.259) were particularly significant.

### 3.4. Interactions and Correlation Analysis of Gut Microbiome

**Figure 4:**
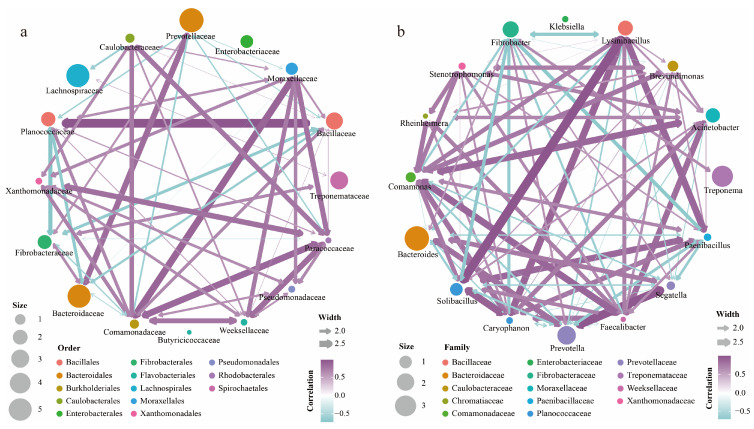
Mapping interaction based on the relative abundance of gut microbiota. (**a**) Gut microbiota interaction network at the family level. Nodes represent different bacterial families, with size indicating relative abundance, color-coded by order. Edge width represents interaction strength, with purple indicating positive correlations and blue-green indicating negative correlations. (**b**) Gut microbiota interaction network at the genus level. Nodes represent different bacterial genera, with size indicating relative abundance, color-coded by family. Edge width represents interaction strength, with purple indicating positive correlations and blue-green indicating negative correlations.

To investigate the relationships among the gut microbiota, we constructed a microbial interaction network based on Spearman correlation. The results showed that at the family level, Planococcaceae and Bacillaceae exhibited a significant positive correlation, while both were negatively correlated with Fibrobacteraceae and Bacteroidaceae. In addition, Moraxellaceae showed a positive correlation with Paracoccaceae, Weeksellaceae, and Comamonadaceae (Figure 4a). Notably, Enterobacteriaceae, Butyricicoccaceae, and Lachnospiraceae did not show correlations with other microbes (Figure 4a), which may reflect their distinct ecological characteristics or specialized survival strategies.

At the genus level, *Lysinibacillus*, *Solibacillus*, and *Paenibacillus* exhibited a significant positive correlation. *Fibrobacter* showed a negative correlation with *Solibacillus*, *Caryophanon*, *Paenibacillus*, and *Lysinibacillus* (Figure 4b). In addition, low-abundance genera such as *Paenibacillus*, *Faecalibacter*, and *Caryophanon* were correlated with various other microbial communities, highlighting their important role in the gut microbial community structure. Notably, *Klebsiella* did not show any significant correlation with other microbes (Figure 4b).

### 3.5. The Relationships Between Diet Diversity and Gut Microbiome Diversity

**Figure 5:**
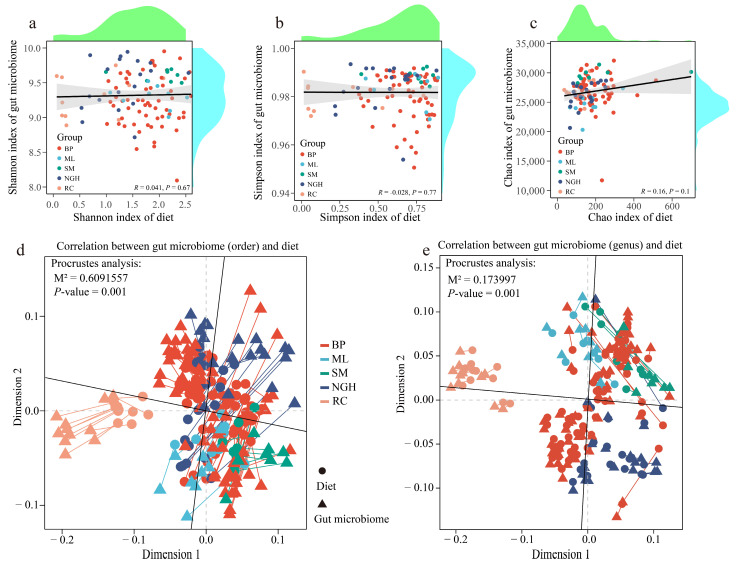
The relationship between gut microbiota diversity and dietary diversity. (**a**–**c**) The relationship between dietary alpha diversity and gut microbiome alpha diversity, with (**a**–**c**) representing the relationships of the Shannon index, Simpson index, and Chao1 index, respectively. Each point represents an individual sample, with different colors corresponding to different groups. The solid line represents the fitted regression line, and the gray shaded area indicates the 95% confidence interval. The marginal density plots display the distribution of dietary and microbiome diversity along the *X*-axis and *Y*-axis, respectively. (**d**,**e**) Procrustes analysis of the correlation between diet and gut microbiota communities, with (**d**) representing the order level and (**e**) representing the genus level of the gut microbiota. Triangles represent the gut microbiota, circles represent diet, and different colors correspond to different groups.

The relationship between dietary diversity and gut microbiome diversity was evaluated using Spearman’s rank correlation analysis. The results revealed no significant correlations between the Shannon, Simpson, and Chao1 indices of the gut microbiota and their corresponding dietary indices (Figure 5a–c and Supplementary Table S11), suggesting that dietary alpha diversity is unlikely to be a primary determinant of gut microbiome diversity. In contrast, Procrustes analysis based on Bray–Curtis distances demonstrated a strong fit and significant correlations between diet and the gut microbiota composition across different taxonomic levels (order levels: M^2^ = 0.6091557, *p* = 0.001; genus levels: M^2^ = 0.173997, *p* = 0.001; Figure 5d,e). These results indicate that diet has an impact on the gut microbiome composition. The PERMANOVA analysis based on microbiome and dietary data showed significant differences between the BP and RC groups, as well as between the RC and SM groups, in both the microbiome and dietary data (Table 1). However, in some groups, the microbiome and dietary data did not show consistent significant differences. This may be due to the fact that the gut microbiome is a complex microecological environment influenced by multiple factors, with diet being only one of the key influencing factors.

### 3.6. The Correlation Between Diet and the Gut Microbiome Community

**Figure 6:**
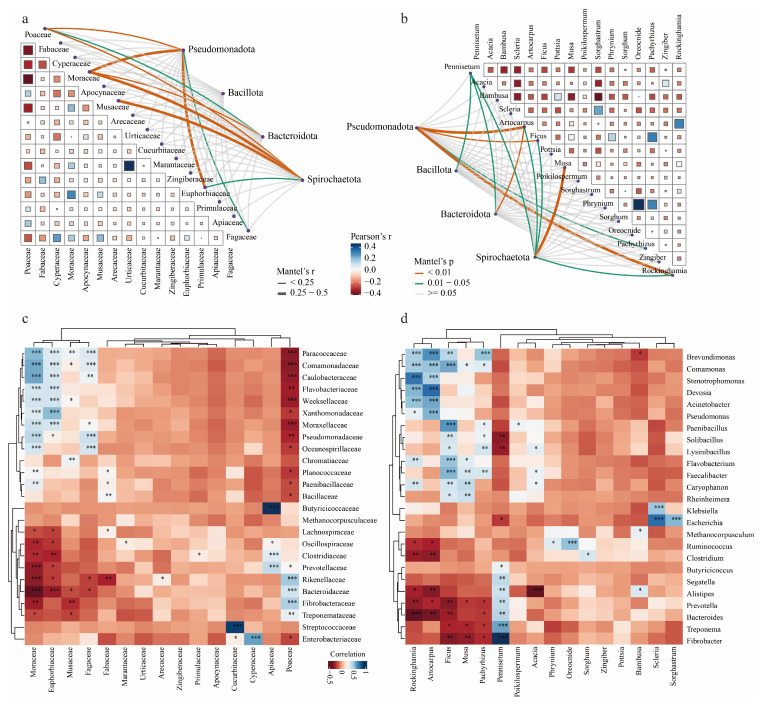
Correlation analysis between gut microbiota and forage plants. (**a**,**b**) Heatmap of Mantel test correlations between the family (**a**) and genus (**b**) levels of the top 16 forage plants and the phylum level of gut microbiota. (**c**,**d**) Pearson correlation analysis of the gut microbiome dataset with the dietary dataset at the family (**c**) and genus (**d**) levels. The color gradient in the heatmap represents the Pearson correlation coefficients between the microbial abundance and dietary data, with blue indicating positive correlations and red indicating negative correlations. Significant differences detected by the Pearson correlation test; “*” indicates significant correlation; * *p* < 0.05, ** *p* < 0.01, and *** *p* < 0.001.

The Mantel test revealed a significant correlation between the abundance of microbial communities at the phylum level and the abundance of different feed plant families and genera. At the family level, Pseudomonadota was correlated with Moraceae and Euphorbiaceae, while Spirochaetota was correlated with Moraceae and Musaceae (Figure 6a). At the genus level, Pseudomonadota was correlated with *Artocarpus* and *Rockinghamia*, and Spirochaetota was correlated with *Musa* (Figure 6b).

Spearman correlation analysis of the relative abundance of diet and gut microbiota at the family and genus levels revealed two food–microbe clusters (Figure 6c,d). At the family level, Poaceae, Moraceae, Euphorbiaceae, and Fagaceae were correlated with various gut microbiota (Figure 6c). Specifically, Apiaceae showed a significant positive correlation with Butyricicoccaceae, while Cucurbitaceae exhibited a positive correlation with Streptococcaceae. At the genus level, *Rockinghamia*, *Artocarpus*, *Ficus*, and *Pennisetum* were correlated with multiple microbes (Figure 6d). Notably, the feed plants *Rockinghamia* and *Artocarpus* were positively correlated with the gut microbiota *Brevundimonas*, *Comamonas*, *Stenotrophomonas*, *Devosia*, and *Acinetobacter* but negatively correlated with *Clostridium*, *Bacteroides*, and *Alistipes*. Additionally, *Acacia* showed a significant negative correlation with *Alistipes*, while *Scleria* and *Sorghastrum* were positively correlated with *Escherichia* (Figure 6d).

## 4. Discussion

In this study, we employed metagenomic sequencing to characterize the gut microbiota composition of different populations of Asian elephants in China and integrated dietary data from corresponding samples to elucidate the interaction between diet and the gut microbiota. The results revealed significant differences in the gut microbiota composition among populations, which are closely associated with their dietary characteristics. These findings not only enhance our understanding of the gut microbiota ecology of Asian elephants but also provide valuable insights for their conservation and food management strategies.

Consistent with previous studies on the gut microbiota of Asian elephants [8,32,33,34], this study found that the gut microbial community was primarily composed of Bacillota and Bacteroidota at the phylum level. These two phyla are widely present in herbivorous wildlife [35,36]. Bacillota can degrade cellulose into volatile fatty acids that are readily available [11,37], while Bacteroidota is an important polysaccharide-degrading phylum capable of breaking down non-fibrous substances [38], providing essential functional support for the normal digestive function of Asian elephants. However, we observed for the first time that Pseudomonadota had a high relative abundance in the SM group (Figure 2a). This may be because previous gut microbiota studies primarily focused on the BP population [32,39,40]. Additionally, the SM population borders Laos, where Asian elephants frequently migrate. The structure and abundance of the gut microbiota significantly differ among populations, suggesting that differences in habitat and diet may be important factors contributing to these microbial variations.

We analyzed the alpha and beta diversity of the gut microbiota across different Asian elephant populations and identified significant differences among populations (Figure 3), with the SM population exhibiting the highest diversity and the RC population the lowest. In general, higher gut microbial diversity is associated with greater compositional complexity and stability, which enhances resilience to external disturbances, environmental adaptability, and the ability to restore homeostasis [41]. These findings suggest that the gut microbiota of the captive RC population may be more vulnerable, with lower adaptability, potentially due to constraints imposed by the captive environment. Furthermore, Spearman correlation analysis revealed complex microbial interactions, particularly among low-abundance genera such as *Paenibacillus*, *Faecalibacter*, and *Caryophanon*, which exhibited significant associations with multiple other taxa (Figure 5). This highlights the potential role of low-abundance taxa in maintaining the gut microbial community structure and function. Therefore, preserving and enhancing gut microbial diversity may be essential for promoting the health and ecological adaptability of Asian elephants.

Through correlation analysis, we found no significant relationship between dietary diversity and gut microbiome diversity (Figure 5a–c), which contrasts with the relationship between dietary diversity and RNA virus diversity in the great evening bat (*Ia io*) [42]. This discrepancy may be attributed to the broad food sources and complex digestive system of Asian elephants. Nevertheless, our Procrustes analysis (Figure 5d,e) and Mantel test (Figure 6) both indicate that food types play a crucial role in shaping the composition of the gut microbiome. These results suggest that while dietary diversity does not directly influence the alpha diversity of the gut microbiome, specific food types may play a crucial role in shaping the microbiome structure by modulating the abundance of particular microbial taxa. In future rescue and rehabilitation efforts, special attention should be given to increasing dietary diversity, particularly incorporating plant species such as Poaceae, Moraceae, Euphorbiaceae, and Fagaceae, as they are significantly correlated with various gut microbiota. Furthermore, the complex relationship between plant species and microbes suggests that when developing refined dietary strategies, the nutritional components of the plants and their impact on the gut microbiota should be considered to enhance the overall health and ecological adaptability of the animals. This finding offers valuable insights into how the dietary preferences of Asian elephants impact their gut microbiome ecology, providing a scientific foundation for optimizing their dietary management strategies.

## Figures and Tables

**Table 1:** Permutational multivariate analysis of variance results for gut microbiome and dietary analysis.

	Gut Microbiome			Dietary		
	R^2^	*p*. Value	*p*. Adjusted	R^2^	*p*. Value	*p*. Adjusted
BP vs. RC	0.10787	0.0001	0.0002	0.22822	0.0001	0.00013
BP vs. SM	0.25908	0.0001	0.0002	0.08952	0.0001	0.00013
BP vs. ML	0.0424	0.0027	0.0030	0.08853	0.0002	0.0002
BP vs. NGH	0.09079	0.0001	0.0002	0.09693	0.0001	0.00013
RC vs. SM	0.4531	0.0001	0.0002	0.63689	0.0001	0.00013
RC vs. ML	0.13787	0.0008	0.0010	0.60108	0.0001	0.00013
RC vs. NGH	0.18319	0.0001	0.00020	0.46086	0.0001	0.00013
SM vs. ML	0.46405	0.0002	0.00033	0.34269	0.0002	0.0002
SM vs. NGH	0.19573	0.0004	0.00057	0.2844	0.0001	0.00013
ML vs. NGH	0.11923	0.0072	0.00720	0.26331	0.0001	0.00013

## Data Availability

Sequence datasets were submitted to the NCBI (National Center for Biotechnology Information) Sequence Read Archive (SRA) and are available under the accession number SUB15228927.

## Supplementary Materials

The following supporting information can be downloaded at https://www.mdpi.com/article/10.3390/genes16050483/s1: Table S1: An overview of the sample collected in this study. Table S2: The abundance data of dietary amplicon sequence variants (ASVs) for each sample. Table S3: The relative abundance data of dietary taxa at the family level for each sample. Table S4: The relative abundance data of dietary taxa at the genus level for each sample. Table S5: Metagenomic sequencing raw data information. Table S6: Metagenomic sequencing raw data information. Table S7: The composition and relative abundance of the gut microbiome at the phylum level across different groups. Table S8: The composition and relative abundance of the gut microbiome at the class level across different groups. Table S9: The composition and relative abundance of the gut microbiome at the order level across different groups. Table S10: The composition and relative abundance of the gut microbiome at the family level across different groups. Table S11: Alpha diversity indices of the gut microbiome and diet.
